# Many Ways to Engage: Uncovering How Adults Navigate a Digitally Presented Health-Related Literacy Item

**DOI:** 10.3390/healthcare14203387

**Published:** 2026-10-11

**Authors:** Gal Kaldes, Sarah E. Miller, Heewon Lee-Laminack, Elizabeth L. Tighe

**Affiliations:** 1 Adult Literacy Research Center, Department of Learning Sciences, Georgia State University, Atlanta, GA 30303, USA; gkaldes1@gsu.edu (G.K.); heewonlee9304@gmail.com (H.L.-L.); 2 Department of Psychology, Georgia State University, Atlanta, GA 30303, USA; smiller154@student.gsu.edu

**Keywords:** digital health literacy, adult literacy, PIAAC, process data

## Abstract

**Background/Objectives:** Digital health literacy refers to the ability to find, understand, and use health information from digital sources. Disparities in these skills contribute to health inequalities and poorer outcomes. This study examines how adults with varying literacy skills navigate a digitally presented health-related literacy task. We identify engagement profiles and examine relations to demographics, daily skills-use, self-reported health, and literacy performance. **Methods:** We analyzed public-use data from the 2022–2023 U.S. Program for the International Assessment of Adult Competencies (PIAAC) Cycle 2 assessment. The sample included 3722 adults who completed a digitally presented medicine-label literacy item. Process indicators included total item time, number of actions, visits, latency before the first action, and interaction speed. Latent profile analysis was used to identify engagement patterns, which were then descriptively examined in relation to demographics, home skills-use, and literacy proficiency. **Results:** Latent profile analysis of total time, actions, visits, hesitation and speed revealed five engagement patterns: Early Exit (8 s, 0 actions, 0% correct; minimal interaction); Hesitant-Minimal (36 s, 2 actions, 30% correct; long pauses with minimal interaction); Rapid Exploratory (54 s, 44 actions, 51%; fast, high-volume clicking); Direct/Routine (57 s, 6 actions, 58%; few actions at a moderate pace); and Deliberate Exploratory (80 s, 20 actions, 62%; longer task time with higher action counts). The identified profiles showed descriptive variation in age, education, self-rated health, skills-use, and literacy level. **Conclusions:** Process data revealed distinct engagement patterns on a digitally presented health-related literacy task that were not captured by accuracy alone. These findings highlight the value of examining how adults interact with digitally presented health-related information, while additional tasks are needed to determine whether similar patterns generalize to broader digital health contexts.

## 1. Introduction

Recent findings from the Program for the International Assessment of Adult Competencies (PIAAC) indicate that approximately 28% of U.S. adults perform below literacy proficiency levels, with an additional 29% nearing but not yet reaching proficiency thresholds [1,2]. These difficulties are associated with barriers to workforce participation, postsecondary success, and navigating increasingly information-rich digital environments [3,4]. Furthermore, adults with developing literacy skills represent a substantial yet understudied population that warrants increasing attention as digital technologies continue to shape employment, education, and access to information [5]. One area of particular importance is digital health literacy, which refers to the ability to locate, understand, and use health information from digital sources [6]. Disparities in these skills may contribute to health inequities and poorer health outcomes [7], yet little is known about how adults with developing literacy skills engage with digital environments in particular digital health settings [8].

The PIAAC and its corresponding framework provide a particularly valuable opportunity to address this gap because they were specifically designed to measure literacy skills across the full adult proficiency spectrum using nationally representative samples [9]. Within the PIAAC framework, literacy is broadly defined as the ability to “access, understand, evaluate and reflect on written texts in order to achieve one’s goals, to develop one’s knowledge and potential and to participate in society” [9,10] (p. 42). Importantly, this definition emphasizes literacy as a construct embedded within authentic daily contexts, including tasks which involve navigating digital health-related information.

A major methodological advancement emerged in PIAAC Cycle 2, in which adults across the full proficiency spectrum completed the literacy assessment digitally on tablets [10]. In earlier cycles, adults identified with limited digital skills were routed to a paper-based version of the assessment rather than completing the digital platform [11,12]. Although this approach was intended to reduce technological barriers, it created a disconnect with the broader PIAAC literacy framework, which conceptualizes literacy as occurring across diverse real-world and increasingly digital contexts [13]. PIAAC Cycle 2 more closely aligns the assessment format with the framework’s conceptualization of literacy itself and creates opportunities to better understand how a broader and more representative range of adults engage with digitally presented health-related information.

Building on the expanded digital framework introduced and administered in PIAAC Cycle 2, the current study aims to better understand how adults engage with digitally presented health-related information. Specifically, we examine whether distinct engagement profiles can be identified from adults’ navigation behaviors on a simulated health literacy item, including time allocation, actions within the digital environment, and interaction patterns. This is important because difficulties with digital health information may reflect not only literacy proficiency, but also how adults interact with and navigate digital information environments [14,15]. Examining these behavioral patterns may therefore provide additional insight into where difficulties emerge during engagement with digitally presented health-related information [16,17,18].

### 1.1. Functional Health Literacy in Digital Contexts

The health-related literacy demands examined in the present study are reflected in the MEDCO unit, a PIAAC literacy task that requires participants to locate, interpret, and apply information presented in medical instructions [9]. Although situated within the literacy domain, successful completion of the task depends on more than reading comprehension alone. Participants must identify relevant information, determine its relevance to the task goal, and interact with information from a digital environment. As such, MEDCO provides a useful context for examining how adults engage with digitally presented health information during task performance.

From broader theoretical perspectives such as the REading as Problem SOLVing (RESOLV) framework [19], these demands extend beyond traditional print literacy because successful task completion requires interactions between the reader, task, and context that influence strategy use, motivation, and self-regulation during engagement. Although RESOLV is not specific to digital environments, digital health tasks may amplify these processes by requiring individuals to navigate digital interfaces while working toward a health-related goal. Similarly, information literacy frameworks such as the Big6 Model [20] and Kuhlthau’s Information Search Process [21] emphasize that successful task performance depends not only on accessing information but also on determining what information is relevant for achieving a particular objective. Difficulties accessing and interpreting health information can have important consequences in everyday contexts. Prior research has linked limited health literacy to lower use of preventive services, greater healthcare utilization, and difficulties with medication management, including interpreting prescription labels and dosage information [22,23,24,25,26,27,28,29,30,31,32,33,34,35,36].

### 1.2. The Role of Process Data in Digital Health Contexts

Although indicators of everyday functioning provide important insight into whether adults successfully complete literacy tasks within a health context, they reveal substantially less about how individuals engage with those tasks and where barriers to successful performance may emerge. Process data, including response times, navigation sequences, clicks, scrolling behaviors, and action patterns captured within digital assessment environments, provide insight into the behavioral processes that unfold as adults work toward a solution within a digital environment [37,38,39,40,41,42]. In digitally mediated health contexts, such indicators may provide insight into how adults search for information, manage uncertainty, and persist through complex healthcare-related tasks. These indicators are particularly relevant within the RESOLV framework [19] because they provide behavioral evidence of how individuals respond to and interact with the demands of a digital task environment while working toward a solution, even when they ultimately arrive at similar performance outcomes.

Emerging research with adult literacy populations further demonstrates the potential value of process data for understanding heterogeneity in digital task engagement that may extend to digital health outcomes. Fang et al. used timing and accuracy data from adults interacting with a digital learning environment and identified four distinct speed-accuracy profiles, which included faster and accurate, slower and accurate, faster with lower accuracy, and slower with lower accuracy [38]. Similarly, Kaldes et al. identified two contrasting time allocation patterns among adults who did not meet literacy proficiency levels (i.e., at or below Level 2 proficiency on PIAAC literacy tasks) [43]. These groups included a high-time subgroup that spent substantially more time per item than adults meeting literacy proficiency (i.e., Levels 3 to 5) and a subgroup that spent substantially less time relative to the group meeting proficiency.

In addition to timing behaviors, action-based indicators offer important insight into how adults interact with digital tasks. Log file metrics such as the total number of clicks, navigation sequences, scrolling behaviors, information revisits, and transitions across interfaces provide insight into how individuals navigate task demands and adapt their behavior during digital problem-solving [39,40,41]. Similar to the heterogeneity observed in timing data, a higher volume of actions does not necessarily reflect a single behavioral pattern. In some contexts, a larger number of actions may indicate strategic exploration and active information seeking, whereas in other contexts it may reflect uncertainty, disorientation, or inefficient trial-and-error navigation. Similarly, fewer actions may reflect either efficient task execution by proficient users or reduced engagement with available tools and information.

Understanding heterogeneity in engagement may help identify behaviors associated with difficulty navigating digitally presented health-related information. This perspective is consistent with adult digital literacy work emphasizing the importance of connecting technology use to meaningful everyday contexts, including health information seeking [44,45,46,47]. Process data may therefore provide a useful way to examine where difficulties emerge during interaction rather than relying on performance outcomes alone.

### 1.3. Current Study

The present study examines heterogeneity in how adults engage with a digitally presented health-related literacy task using process data from PIAAC Cycle 2. Prior research using process data has demonstrated that adults are not behaviorally homogeneous during digital task engagement and that distinct interaction patterns may emerge even among individuals with similar performance outcomes [38,43]. Although prior findings have highlighted substantial variation among adults with lower literacy proficiency, health-related information and digital health tasks are encountered by adults across the literacy spectrum, making it important to examine engagement patterns more broadly. However, relatively little research has examined how adults behaviorally engage with digitally presented health-related literacy tasks specifically. To address this gap, the present study extends prior process data research by applying latent profile analysis (LPA) to multiple time- and action-based indicators from a PIAAC health-related literacy item in a sample spanning the full literacy proficiency spectrum. This approach allows us to examine whether adults with similar task outcomes nevertheless show different combinations of timing and interaction behaviors within the same health-related literacy task. Specifically, the study examines patterns across multiple process indicators, including actions, visits, latency share, speed, and total time, to characterize how adults navigate and engage with the task.

The focus on a single health-related item reflects both conceptual and methodological considerations associated with exploratory process data analyses. Process patterns are shaped not only by participants’ strategies and engagement behaviors, but also by variation in item structure, difficulty, scoring rules, and interface demands across tasks [48]. Restricting analyses to a single item minimizes heterogeneity attributable to differences in task design and allows interaction patterns to be interpreted within a common digital environment. This consideration is particularly important in person-centered analyses because latent engagement profiles may otherwise reflect differences across tasks rather than meaningful differences in how adults interact with the same task environment. At the same time, the selected item reflects a health-related digital context involving interaction with healthcare-related information, situating the task within broader questions about digital health literacy and digitally mediated healthcare access. Given that PIAAC Cycle 2 currently includes a limited number of directly comparable health-related literacy items presented digitally, the present study is intended as an exploratory first step toward understanding engagement with digitally presented health-related information.

The study additionally examines how the identified engagement profiles are characterized by demographic characteristics, self-reported health, skills-use practices at home, and overall literacy proficiency. Prior PIAAC research has demonstrated variation in both literacy performance and digital task engagement across demographic and skills-use characteristics [43,49]. We therefore included these variables to provide an exploratory description of how the engagement profiles are situated within broader patterns of adult experiences and literacy practices.

We ask the following research questions:

(1) Which engagement profiles emerge from process data indicators (e.g., total time on task, number of actions) during a PIAAC health-related literacy item?
(2) How are the identified engagement profiles characterized by demographics (age, education, self-rated health), skills-use practices at home (information and communication technology [ICT], numeracy, reading, writing), and overall literacy performance?

## 2. Methods

This study used data from the United States public use Program for the International Assessment of Adult Competencies (PIAAC) Cycle 2 dataset [50] to examine how adults engage with a digitally presented health-related literacy task. PIAAC is an international large-scale assessment of adult skills administered across two cycles (Cycle 1: three waves; Cycle 2: one wave) designed to evaluate proficiencies considered essential for participation in contemporary society. Cycle 2 (2022–2023) was administered to nationally representative samples of adults across participating countries and assessed cognitive domains including literacy, numeracy, adaptive problem-solving, and reading and numeracy components [2].

The present study focused specifically on process data generated during interaction with a health-related literacy item from the MEDCO unit within the U.S. sample. In addition to process indicators derived from the digital assessment environment, the study incorporated literacy proficiency estimates from the assessment as well as demographic, self-reported health, and everyday skills-use variables obtained from the background questionnaire to examine broader patterns associated with engagement profiles. The following sections describe the analytic sample, literacy domain, focal literacy item, process indicators, and background questionnaire measures included in the analyses.

### 2.1. Participants

Data were available for 3765 respondents in the U.S. PIAAC Cycle 2 public use file [50]. The present analyses were restricted to 3722 participants with complete process data for the MEDCO Question 2 across all proficiency levels. The 43 excluded respondents were missing the process variables required for the present analyses. Descriptively, these respondents had lower average literacy proficiency than those retained in the analytic sample (*M* = 185.7 vs. 258.9), suggesting that the analytic sample may underrepresent adults with particularly low literacy proficiency. Our analytic sample comprised 53% female participants, 94% monolingual participants, and 18% of participants born outside the U.S. Of the sample, 47% completed high school or less, 18% completed some college, and 35% held a bachelor’s degree or higher (Please note race and ethnicity variables were not available in the U.S. PIAAC Cycle 2 public use dataset examined in the present analyses. Readers interested in nationally representative racial and ethnic distributions within the broader PIAAC sample are referred to NCES summary reports and accompanying technical documentation [3]).

### 2.2. PIAAC Literacy Domain

According to the PIAAC Cycle 2 literacy framework, literacy involves “accessing, understanding, evaluating, and reflecting on written texts to achieve one’s goals, develop one’s knowledge and potential, and participate in society” [10] (p. 42). The literacy assessment included a wide range of authentic text formats and task contexts designed to reflect the demands adults encounter in everyday life, including continuous, noncontinuous, and mixed digital texts presented across personal, workplace, community, and educational settings. Literacy tasks assessed multiple cognitive processes, including accessing information, understanding and integrating ideas across texts, and evaluating the relevance and credibility of information sources. The literacy assessment includes 80 literacy items distributed across varying levels of difficulty and cognitive demands, with respondents completing adaptively assigned subsets of items based on their performance.

Literacy proficiency was reported on a 0–500 scale organized into proficiency levels ranging from Below Level 1 through Level 5, with higher levels reflecting increasingly complex reading and information processing skills. Adults performing Below Level 1 (below 176 points) typically demonstrate difficulty locating and using information in simple texts, particularly when information must be inferred or integrated across multiple pieces of information. Adults performing at Level 1 (176–225 points) can generally locate explicitly stated information in relatively short and simple texts but may struggle with competing information and more complex integration demands. Adults performing at Level 2 (226–275 points) are typically able to identify and interpret information in moderately simple texts but may continue to have trouble evaluating relevance, integrating information across sources, and navigating digitally mediated materials. Adults performing at Level 3 (276–325 points) are generally able to integrate information across texts and apply more complex reasoning strategies to accomplish literacy tasks. Adults performing at Level 4 (326–375 points) and Level 5 (376 points and above) are increasingly able to evaluate credibility, synthesize information across multiple sources, and engage with complex and abstract literacy tasks within digitally mediated environments [9]. Within the PIAAC framework, adults performing at or below Level 2 are generally considered not to have reached literacy proficiency levels needed to consistently navigate and use complex written information in contemporary society, particularly within digitally mediated environments [1,9]. Based on weighted literacy proficiency estimates, approximately 11% of adults performed Below Level 1, 16% at Level 1, 28% at Level 2, 32% at Level 3, 12% at Level 4, and 1% at Level 5.

### 2.3. MEDCO Item

**Figure 1:**
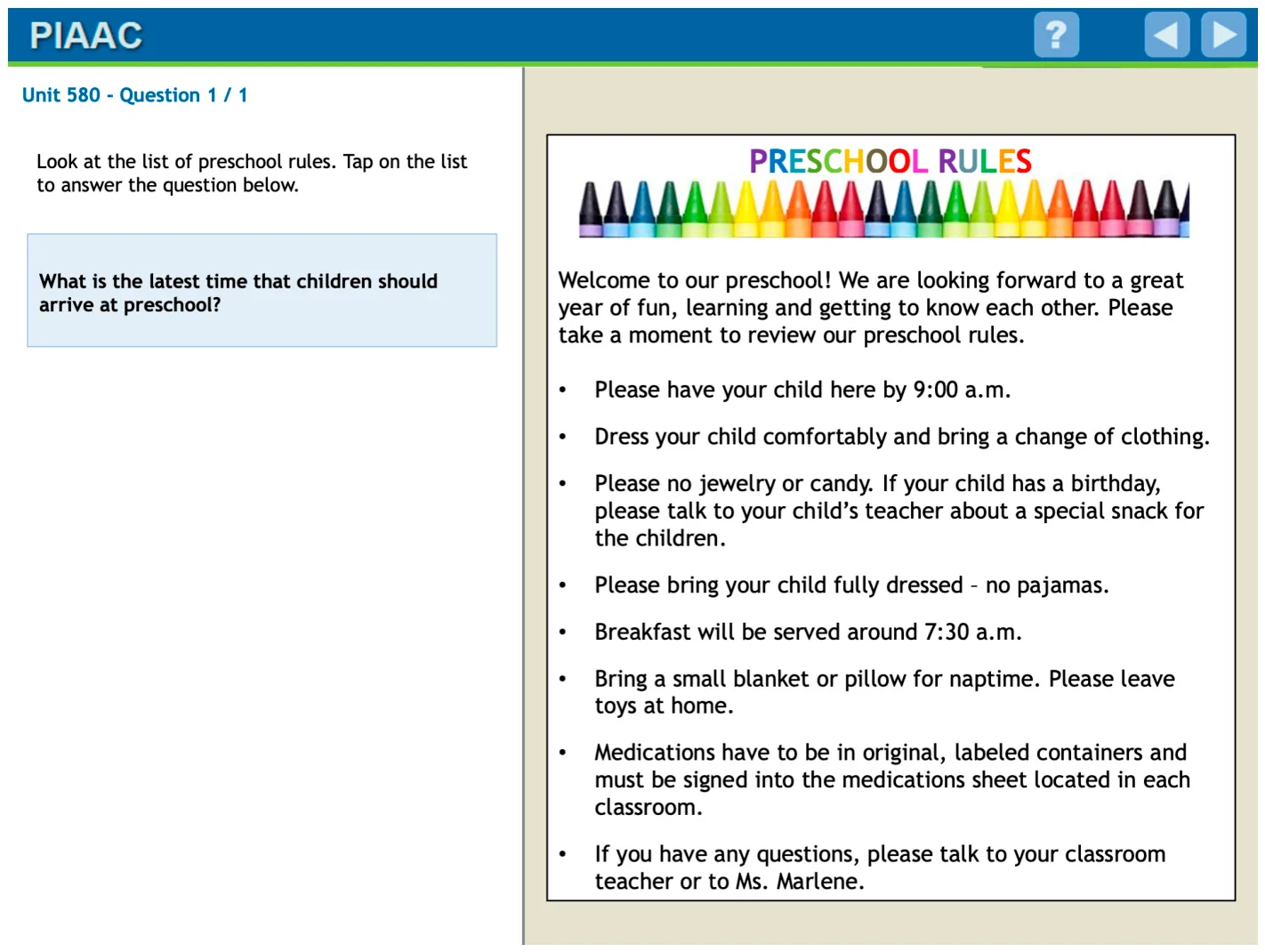
Illustrative Example of the PIAAC Digital Literacy Interface. Note. Figure 1 is provided to illustrate the general appearance and interaction format of the PIAAC digital literacy interface; it is not the MEDCO item analyzed in the present study. The screenshot is adapted from a publicly released PIAAC literacy item. In the MEDCO task, participants reviewed medication instructions presented within the digital interface and used a highlighting tool to identify information relevant to a prescribed dosage schedule. The MEDCO task materials are restricted and therefore cannot be presented here.

The focal literacy task analyzed in the present study was the MEDCO Question 2, a PIAAC literacy item that assesses reading comprehension within a health context. The task required respondents to review digitally presented health-related information and identify relevant portions of text by highlighting information associated with a specific question or prompt. Because the MEDCO task materials are restricted and cannot be reproduced, Figure 1 presents a publicly released PIAAC literacy item to illustrate the general appearance of the digital assessment interface and the type of highlighting interaction used in the MEDCO task. The displayed item is not the MEDCO task analyzed in the present study.

The MEDCO task was selected because it represented one of the few publicly available PIAAC literacy items that specifically focused on health-related information. MEDCO Question 2 was selected over Question 1 because it demonstrated substantially greater variability in participant performance and engagement behaviors. Whereas approximately 88% of participants answered Question 1 correctly, only approximately 50% answered Question 2 correctly. Participants additionally demonstrated greater overall interaction levels on Question 2, averaging approximately 9.7 item actions compared to 7.8 item actions on Question 1. These patterns suggest that Question 2 provides greater discrimination across participants and greater potential for identifying heterogeneous engagement patterns during interaction with the digitally presented health-related literacy task.

To examine these engagement patterns more directly, the present study analyzed multiple publicly available process indicators capturing participants’ interaction behaviors during task completion. These indicators included the total number of actions performed during item interaction (D307402A), total time spent on the item in milliseconds (D307402TT), number of visits to the item screen (D307402V), and latency to first action (D307402F). Two additional indicators were derived to represent the relative timing and pace of participants’ interaction with the task. Latency share was calculated as the proportion of total item time elapsed before the first recorded action (D307402F/D307402TT), providing a relative measure of delay before interaction. For respondents who made no recorded actions, the public use first action latency value was coded as zero, resulting in a latency share of zero in the primary specification; an alternative treatment of these cases was examined in sensitivity analyses. Finally, action speed was calculated as the number of item actions per second (D307402A/D307402TT), providing a measure of interaction pace. Because these derived indicators are mathematical functions of other process variables included in the profile model, additional sensitivity analyses examined whether the substantive profile structure depended on their inclusion.

### 2.4. Background Questionnaire

PIAAC Cycle 2 includes an extensive background questionnaire designed to capture respondents’ demographic characteristics, educational experiences, labor market participation, health, and everyday skills-use practices [50]. The present study used selected background questionnaire variables to examine demographic and skills-use variation across latent engagement profiles. Demographic variables included age group (AGEG10LFS), educational attainment (B2_Q01), and self-rated health (I2_Q03). The original PIAAC age categories were collapsed into three broader groupings representing younger (18–34 years), middle-aged (35–44 years), and older (45+ years) adults to improve interpretability and reduce sparsity across latent profiles. Educational attainment categories based on International Standard Classification of Education (ISCED) levels were similarly collapsed into three broader categories representing high school or less (ISCED 0–3), some college or sub-baccalaureate education (ISCED 4–5), and bachelor’s degree or higher (ISCED 6–8). Self-rated health was retained using the original PIAAC response categories ranging from *poor* to *excellent*. Bilingual status and employment indicators were not included in the primary profile comparisons due to limited variability in bilingual status (approximately 6% bilingual) and substantial routing-related missingness in the employment variable (approximately 41% valid skips).

Skills-use variables were selected to capture everyday information and communication technology (ICT), numeracy, reading, and writing practices within home contexts, including ICT use at home (ICTHOMEC2), numeracy at home (NUMHOMEC2), reading at home (READHOMEC2_T1), and writing at home (WRITHOMEC2). These variables represent PIAAC-derived indices based on multiple self-report questionnaire items assessing the frequency with which adults engage in technology-, numeracy-, reading- and writing-related, activities in everyday life. For example, respondents report how often they engage in activities such as performing calculations, reading prose and document texts, or using digital technologies to access information, with response options ranging from *never* to *every day*. The derived indices aggregate responses across multiple items and are reported on continuous scales, with higher values indicating more frequent engagement in the corresponding activities. Skills-use at home variables were prioritized because workplace skills-use variables contained substantially larger proportions of routed or nonapplicable responses across domains (*M* = 24.7%) relative to skills-use at home variables (*M* = 1.0%). This pattern likely reflected participants who were not currently employed or not engaged in workplace literacy activities.

### 2.5. Data Analytic Approach

Latent profile analyses (LPAs) were conducted using Gaussian mixture modeling implemented with the mclust package [51]. The primary LPA was unweighted because the goal was to identify behavioral engagement patterns within the analytic sample rather than estimate population prevalence. Models used the EEI parameterization, which specifies diagonal covariance matrices with equal volume and shape across profiles. The primary model included five indicators: total item actions, total item time, number of item visits, latency share, and action speed. Time-based variables were winsorized at the 99.5th percentile, highly skewed indicators (actions, total time, visits, and action speed) were log-transformed, and all indicators were standardized before model estimation.

Models containing three through ten profiles were estimated. Model selection considered statistical fit, classification quality, profile size, and substantive interpretability. Candidate solutions were required to have entropy ≥ 0.75 and a smallest profile containing at least 3% of the analytic sample. Because there is no universally accepted minimum profile size cutoff, we selected 3% as a pragmatic threshold to reduce the likelihood of retaining solutions defined by very small profiles, which may be less precise and difficult to interpret substantively [52]. In the present sample, this threshold corresponded to approximately 112 participants, allowing relatively uncommon engagement patterns to be retained while excluding very small higher-order profiles. Among eligible solutions, the model with the largest Bayesian Information Criterion (BIC) was preferred; for BIC and Integrated Completed Likelihood (ICL) values produced by mclust, larger values indicated better fit, including when values were negative. Neighboring solutions were also examined for substantive interpretability and evidence of small or overly subdivided profiles.

Sensitivity analyses examined whether the profile structure depended on indicator selection or coding (see Supplementary Text S1; Table S1). Because latency share and action speed were derived from other process variables and visits showed little variability (Table S2), alternative specifications excluded action speed, visits, or both. Additional analyses removed latency, varied its coding for participants with no recorded actions, substituted first action/exit time, or used raw first action time among participants who acted. The primary five-indicator model was also re-estimated using the PIAAC final respondent sampling weight (SPFWT0). Model selection followed the same criteria as the primary analysis. Correspondence with the primary classification was summarized using the Adjusted Rand Index (ARI), with values closer to 1 indicating greater agreement and values near 0 indicating little agreement beyond chance. ARI was interpreted continuously and considered alongside the number, size, and substantive patterning of the profiles. Because these analyses were robustness checks, RQ2 analyses were based on the primary five-indicator, five-profile solution.

For RQ2, participants were assigned to their most likely profile based on posterior classification probabilities. Demographic percentages and skills-use summaries were survey weighted using SPFWT0, with confidence intervals for skills-use means estimated using the 80 replicate weights (SPFWT1–SPFWT80) and Fay’s balanced repeated replication (BRR). As a sensitivity check for classification uncertainty, these summaries were also recomputed using posterior membership probabilities rather than most likely profile assignments (Table S3).

Literacy proficiency estimates incorporated all 10 plausible values (PVLIT1–PVLIT10), SPFWT0, and the 80 replicate weights using Fay’s BRR procedures. Estimates and confidence intervals were calculated separately across plausible values and combined following OECD and NCES analytic recommendations [1,2]. Because profile estimation was unweighted, profile proportions should not be interpreted as nationally representative prevalence estimates. RQ2 was intended to characterize the profiles descriptively rather than test between-profile differences; therefore, we did not conduct pairwise significance tests or calculate standardized effect sizes for those comparisons. Instead, the magnitude of the observed differences is presented directly through survey-weighted percentages, means, and 95% confidence intervals.

## 3. Results

Preliminary descriptive analyses indicated substantial variability across the five process indicators used in the latent profile analyses (Table 1). The indicators captured different aspects of task engagement, including overall interaction levels (actions), persistence within the task environment (total time), revisiting behavior (visits), hesitation prior to engagement (latency share), and interaction pace (action speed). Latent profile models ranging from three to ten profiles were subsequently estimated. Models containing three, four, and five profiles satisfied the minimum-profile-size (≥3%) and entropy (≥0.75) criteria described above. Among these models, the five-profile solution had the largest BIC. Although BIC and entropy continued to improve for some higher-order solutions, the six-profile model introduced a profile containing only 10 participants (0.3% of the analytic sample), with still smaller profiles emerging in subsequent solutions (Table 2). The five-profile solution was therefore retained as the primary model based on the combined consideration of statistical fit, classification quality, profile size, and substantive interpretability. Additional comparisons with neighboring solutions and alternative indicator specifications are reported in Table S1.

### 3.1. RQ1: Engagement Profiles

**Figure 2:**
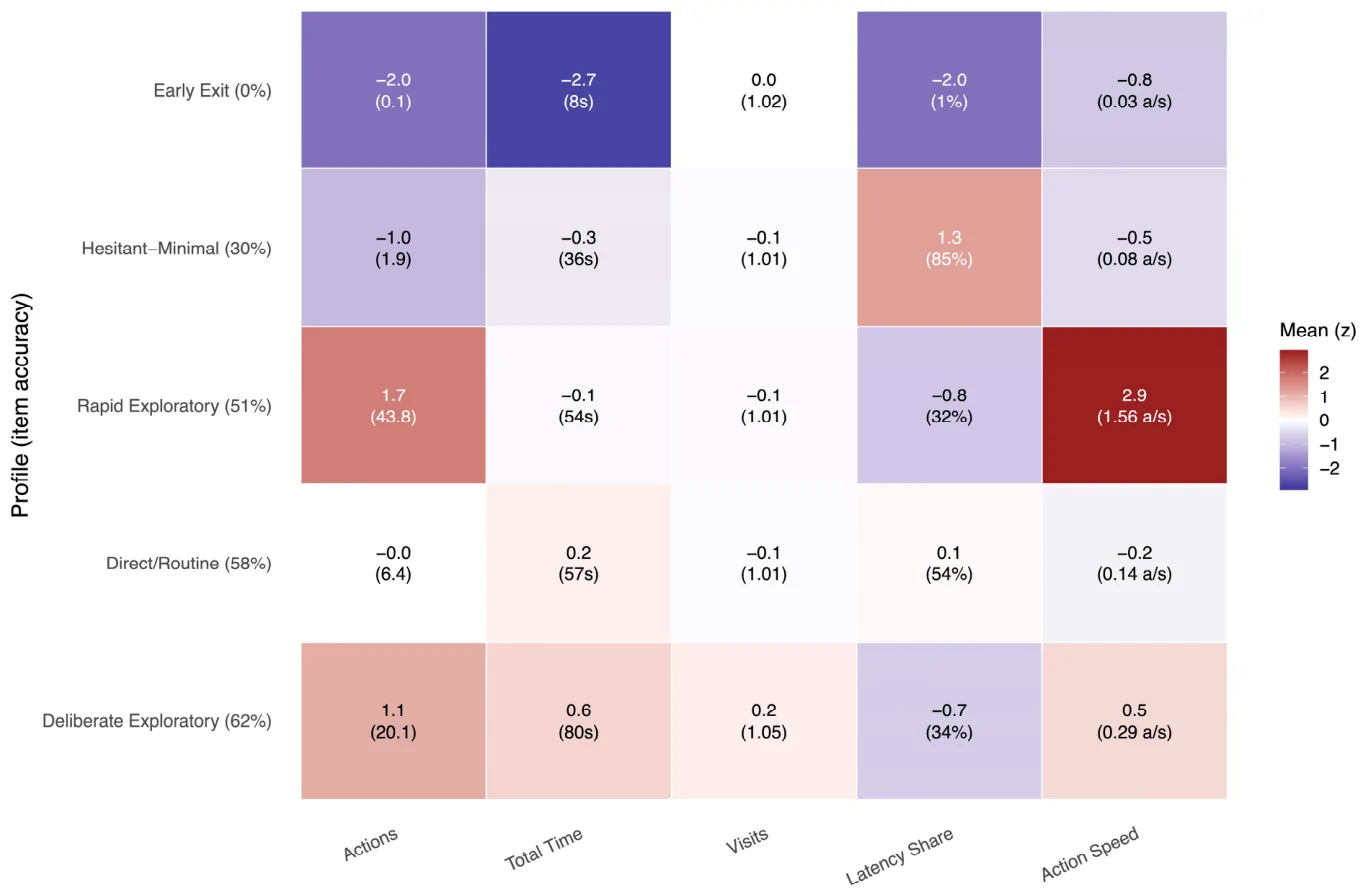
Heat Map of Process Indicators by Profile. Note. Cell values represent profile-specific standardized means (z-scores), with raw metric means shown in parentheses. Positive values indicate above-average levels of the indicator relative to the full sample, whereas negative values indicate below-average levels. Percentages shown in profile labels represent the proportion of participants who correctly completed the MEDCO task. Actions, total time, visits, and action speed were log transformed prior to latent profile analyses. The heatmap illustrates that successful task performance emerged through multiple patterns of engagement, whereas lower performance was associated with distinct forms of reduced interaction and disengagement.

Figure 2 illustrates the standardized process data patterns associated with each latent profile, with original scale means provided to facilitate interpretation. The profiles varied across the process indicators and item accuracy, ranging from minimal engagement to more sustained forms of interaction. The five identified profiles were labeled Early Exit, Hesitant-Minimal, Rapid Exploratory, Direct/Routine, and Deliberate Exploratory (Table 3). The Early Exit profile showed almost no interaction with the task and very little time on the item, whereas the Hesitant-Minimal profile showed relatively few actions but the highest latency share. The Rapid Exploratory profile was characterized by the highest number of actions and fastest interaction pace, whereas the Direct/Routine profile showed fewer actions and a slower pace. The Deliberate Exploratory profile combined relatively high action counts with the longest average task time and the highest item accuracy.

### 3.2. Sensitivity Analyses

Sensitivity analyses generally supported the broader engagement patterns observed in the primary model, although the exact number and composition of profiles varied across alternative specifications (Table S1). Removing action speed largely combined the Rapid Exploratory and Deliberate Exploratory profiles, suggesting that interaction pace contributed to distinguishing these patterns. Visits also showed limited variability, with 98.3% of participants visiting the item once (Table S2). Excluding visits favored a seven-profile solution; however, a five-profile solution under the same reduced indicator specification also met the prespecified criteria and showed substantial correspondence with the primary classification (ARI = 0.78). This suggests that removing visits permitted additional subdivision of the profiles without eliminating the broader engagement patterns identified in the primary model.

Results were also sensitive to how latency was represented for participants who made no recorded actions. However, removing latency entirely again recovered the same five substantive engagement patterns, and the sampling-weighted model reproduced the primary solution with highly similar classifications (ARI = 0.92). Overall, these analyses support the broader profile structure while suggesting that the distinction between Rapid Exploratory and Deliberate Exploratory should be interpreted more cautiously. Additional details regarding the sensitivity analyses and their interpretation are provided in Supplementary Text S1 and Table S1.

### 3.3. RQ2: Demographic, Skills-Use, and Literacy Characteristics of the Profiles

**Figure 3:**
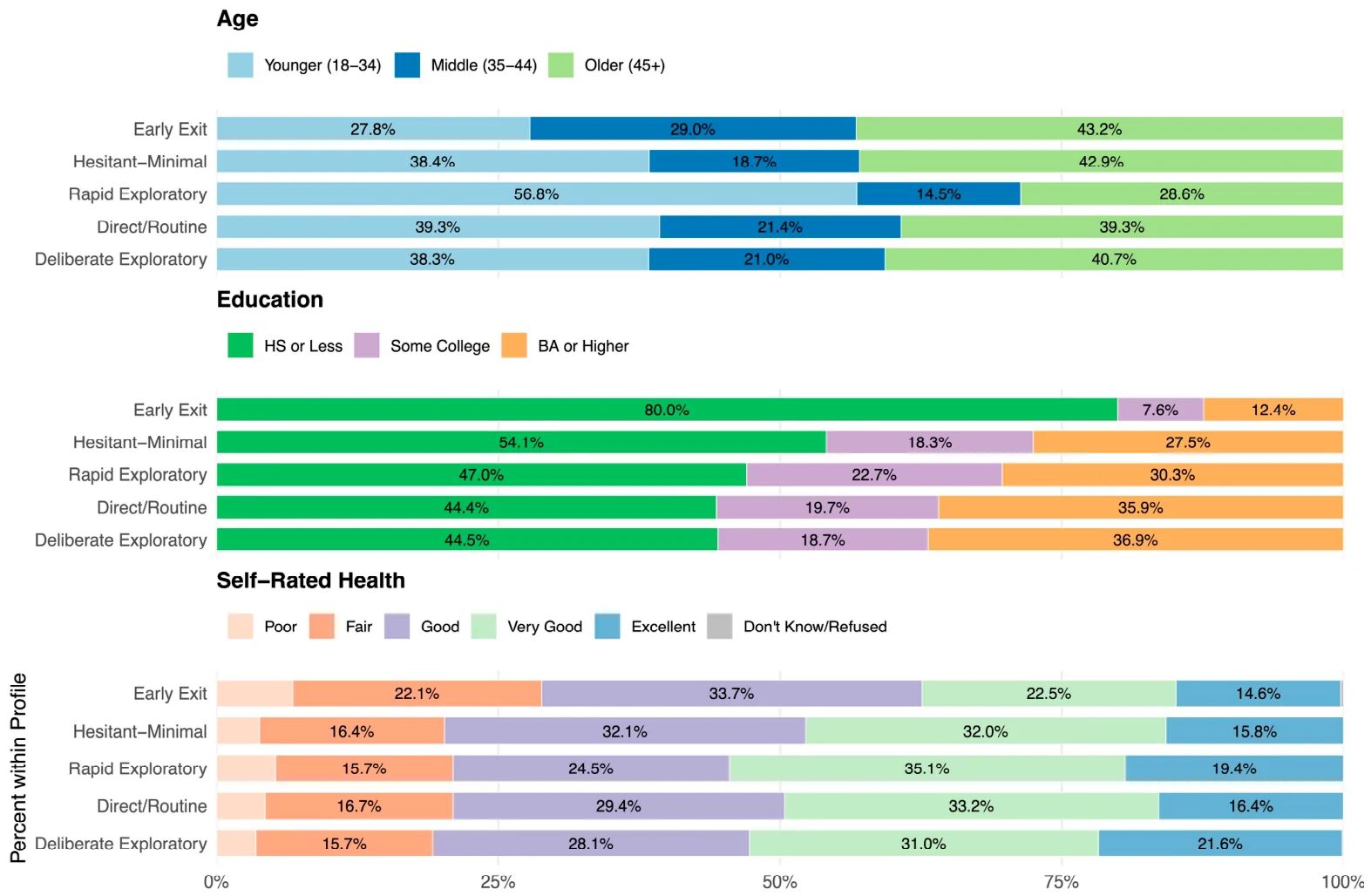
Age, Education, and Health Status Demographics by Profile. Note. Bars represent survey-weighted within-profile percentages using the PIAAC final respondent sampling weight (SPFWT0). Percentages may not sum to exactly 100% because of rounding. Percentage labels are shown within segments where space permits; complete percentages for all categories are reported in Table 4.

The five identified profiles also showed descriptive variation in demographic characteristics (Table 4, Figure 3). The most pronounced pattern was observed for educational attainment, with the Early Exit profile containing the largest proportion of participants with a high school education or less (80%), whereas approximately 36–37% of participants in the Direct/Routine and Deliberate Exploratory profiles held a bachelor’s degree or higher. The Rapid Exploratory profile contained the largest proportion of younger adults (57%), while variation in self-rated health was comparatively smaller.

**Figure 4:**
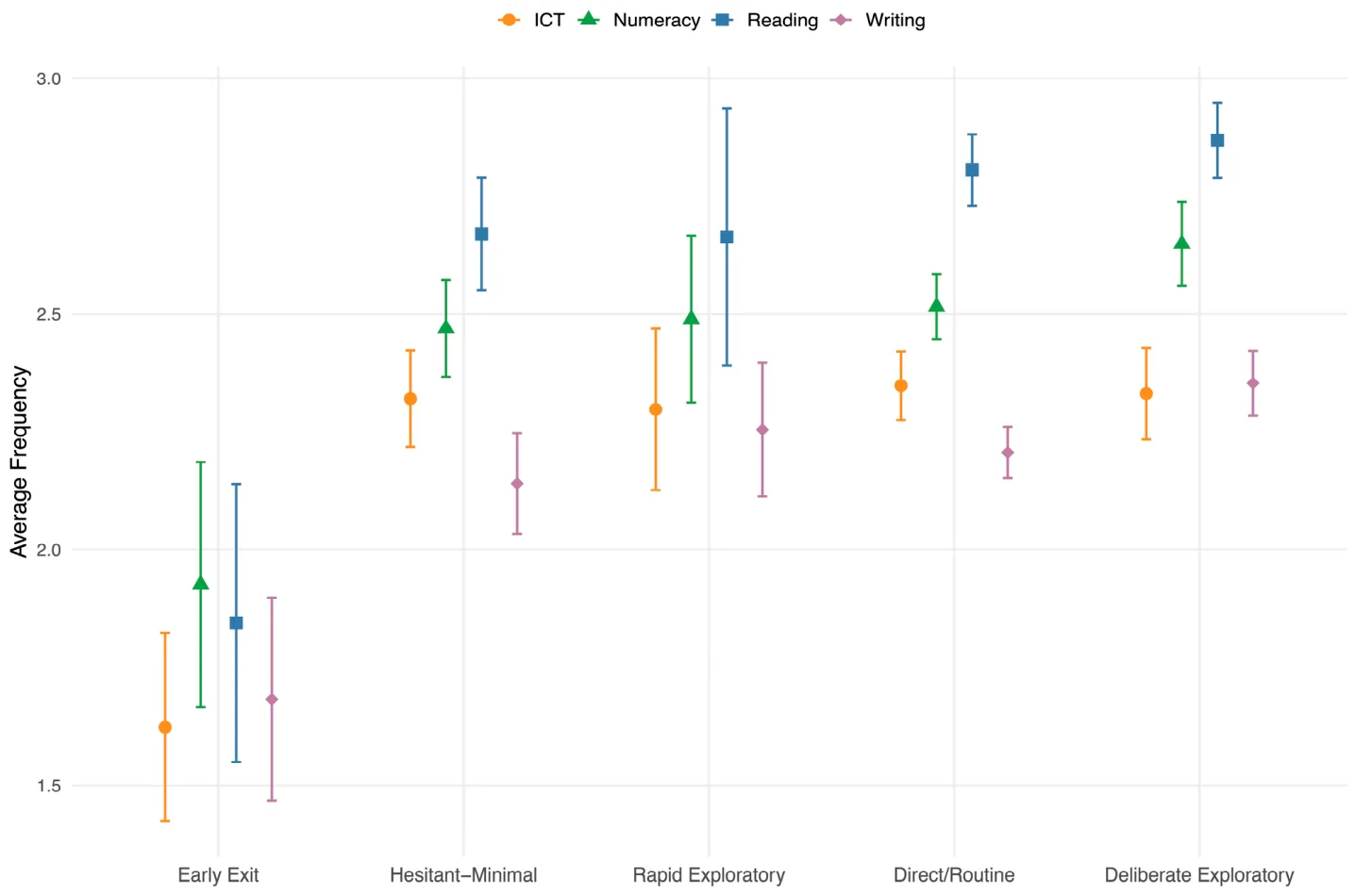
ICT, Numeracy, Reading, and Writing Skills-Use at Home by Profile. Note. Points represent survey-weighted mean scores on the PIAAC Cycle 2 home skills-use indices for ICT, numeracy, reading, and writing. Error bars represent 95% confidence intervals estimated using the 80 replicate weights and Fay’s balanced repeated replication procedures. Higher scores indicate greater self-reported use of the corresponding skill domain.

Descriptive variation also emerged across ICT, numeracy, reading, and writing skills-use at home (Table 5, Figure 4). The Early Exit profile reported the lowest average skills-use across all four domains. In contrast, the Direct/Routine and Deliberate Exploratory profiles generally reported the highest levels of reading and numeracy skills-use at home, whereas differences in writing and ICT skills-use were comparatively smaller. The Rapid Exploratory profile generally showed intermediate levels of skills-use across domains.

**Figure 5:**
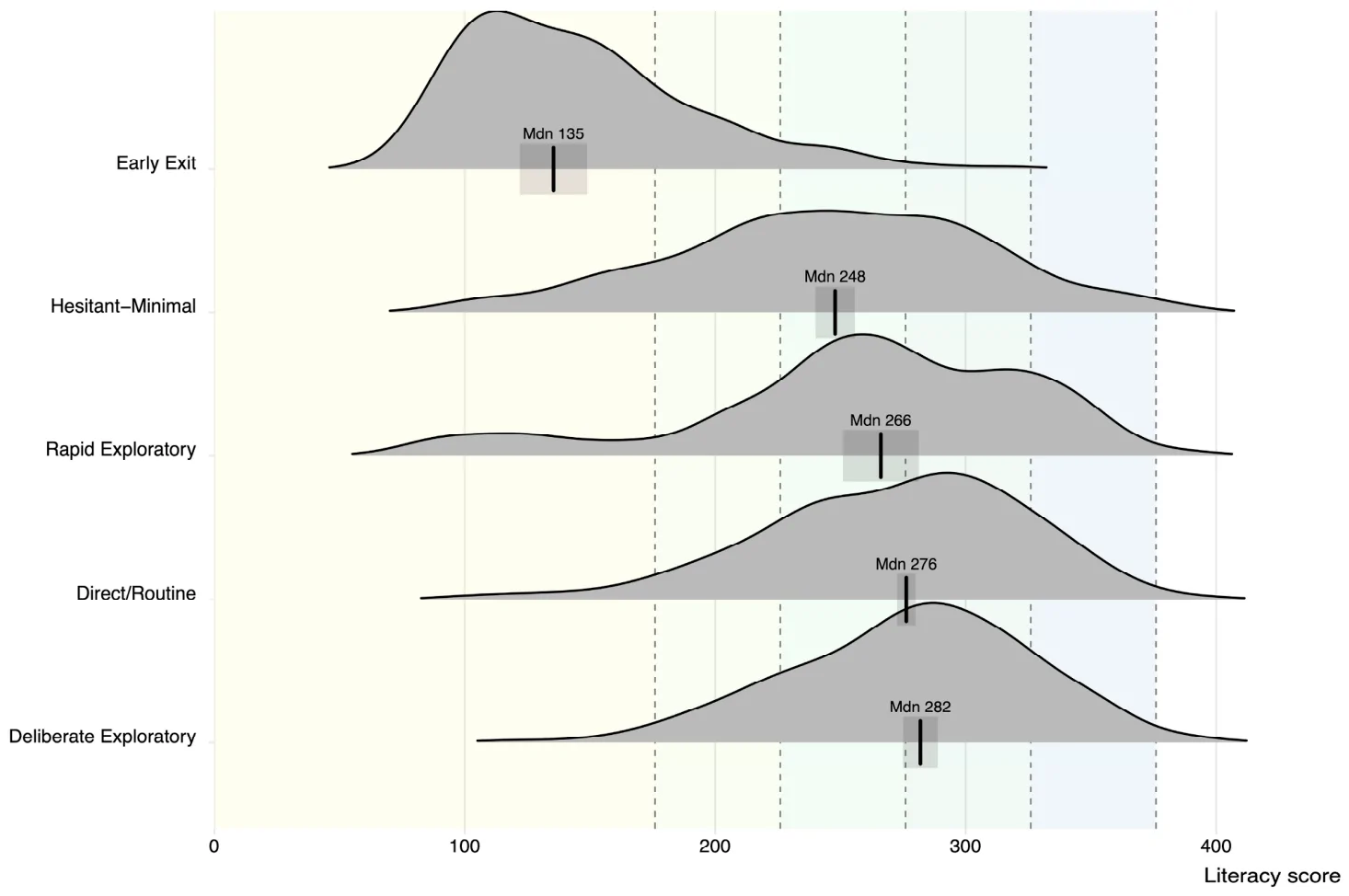
Literacy Score Distribution by Profile. Note. Density ridges represent weighted distributions of literacy proficiency scores (PVLIT) within each engagement profile using the PIAAC final sampling weight (SPFWT0). Vertical dashed lines and shaded regions denote PIAAC literacy proficiency levels. Black vertical lines indicate profile-specific median literacy scores, and shaded rectangles represent 95% confidence intervals around the median. Median estimates and confidence intervals were calculated by combining all literacy plausible values (PVLIT) and replicate weights using Fay’s balanced repeated replication method.

Finally, the profiles descriptively varied in literacy performance (Table 5 and Table 6, Figure 5). Average literacy scores increased from 144.79 in the Early Exit profile to 278.15 in the Deliberate Exploratory profile. Seventy-eight percent of participants in the Early Exit profile performed below Level 1, whereas the Hesitant-Minimal and Rapid Exploratory profiles were centered within Level 2 and the Direct/Routine and Deliberate Exploratory profiles within Level 3. Together, these findings show a descriptive progression in literacy performance across the engagement profiles, with more successful patterns of task engagement generally associated with higher literacy proficiency. Because MEDCO Question 2 is itself part of the PIAAC literacy assessment, these associations should not be interpreted as evidence that the engagement profiles are independent of literacy proficiency.

As a sensitivity check, we repeated the survey-weighted demographic, skills-use, and literacy summaries using posterior class probabilities rather than most likely profile assignments. Results were highly similar, with maximum differences of 1.7 percentage points for demographic characteristics, 0.03 points for skills-use means, 2.4 points for mean literacy proficiency, and 1.4 percentage points for literacy-level distributions (Table S3). We therefore retained the primary profile assignments for the descriptive RQ2 analyses.

## 4. Discussion

In the present study, we examined heterogeneity in engagement with a digitally presented health-related literacy task by identifying latent engagement profiles using both time- and action-based process indicators. Findings demonstrated that examining these indicators in combination revealed several meaningful patterns in how participants with varying literacy skills engaged with the MEDCO task. Sensitivity analyses generally supported the broader engagement patterns observed in the primary model, although the finer distinction between Rapid Exploratory and Deliberate Exploratory engagement was more sensitive to the indicators included in the model.

Two profiles demonstrated relatively low levels of interaction with the task environment and lower accuracy overall. The Early Exit profile was characterized by a near complete lack of interaction, with minimal actions and very little time spent in the item environment. In contrast, the Hesitant-Minimal profile also demonstrated few actions but spent substantially more time in the item before interacting with the environment. This pattern may reflect time spent reading through the materials, orienting to the interface, or some combination of these processes.

Three additional profiles reflected comparatively higher levels of engagement within the digital environment, which included Rapid Exploratory, Direct/Routine, and Deliberate Exploratory. The Rapid Exploratory profile demonstrated the highest rate of actions within a moderate amount of time and relatively limited hesitation before interacting with the environment. In contrast, the Direct/Routine and Deliberate Exploratory profiles demonstrated the highest accuracy overall but differed in their interaction patterns across time and actions. The distinction between Rapid Exploratory and Deliberate Exploratory engagement should be interpreted somewhat cautiously because sensitivity analyses indicated that these patterns were more likely to combine when action speed was not included as a profile indicator.

Consistent with Kaldes et al. [43], the identified engagement profiles also showed descriptive variation across demographic characteristics, skills-use practices, and overall literacy performance. Profiles characterized by more sustained engagement and higher task accuracy generally demonstrated higher educational attainment, stronger literacy performance, and greater reading and numeracy skills-use at home, whereas profiles characterized by lower engagement and lower accuracy tended to demonstrate lower educational attainment, lower ICT skills-use, poorer self-rated health, and lower overall literacy proficiency. At the same time, these associations should be interpreted in light of the fact that MEDCO is itself a literacy assessment item, and literacy proficiency is therefore closely connected to the demands of the focal task.

Additional variation also emerged across age groups, particularly within the Rapid Exploratory profile, which was characterized by younger adults and substantial heterogeneity in accuracy. Collectively, these findings are broadly consistent with prior research linking literacy proficiency, everyday literacy practices, and digital engagement to health-related outcomes and participation in digitally mediated environments [22,53,54]. However, the present findings suggest that these broader differences may also be reflected in how participants engage with a digitally presented health-related literacy task. The following sections consider how these task-specific patterns relate to prior research on engagement with digital health information.

### 4.1. Interpreting Engagement Profiles in the MEDCO Task

Because the process indicators capture observable interaction behaviors rather than participants’ cognitive processes or motivations, the interpretations below are offered as possible explanations for the observed patterns rather than direct evidence of why those behaviors occurred. From a theoretical perspective, the heterogeneity observed across the identified engagement profiles aligns closely with models suggesting that engagement within digital health environments is shaped by the relationship between the reader and the demands imposed by the information environment itself. RESOLV and related information literacy frameworks [19,20,21] conceptualize engagement as a dynamic process in which readers continuously make decisions about what information deserves attention and how that information should be interpreted while progressing through a task. Within digital health contexts, this process may become especially difficult when individuals encounter unfamiliar terminology, dense written materials, or digital layouts that require sustained navigation through health-related content [22,32,35,36]. These theories therefore suggest that variability in engagement patterns should be expected because adults may approach the same health information environment in fundamentally different ways. The identified profiles may reflect different ways participants responded to the demands of the MEDCO task.

The clearest interpretation likely applies to the Early Exit profile. Given the combination of minimal time, almost no interaction with the environment, and consistently low accuracy, this pattern may reflect disengagement before meaningful processing of the health-related information occurred. In educational measurement, such rapid, low interaction sequences are commonly interpreted as indicators of rapid responding, disengagement, or immediate task abandonment, in which users terminate interaction before fully processing task information [17,39,40]. In a real-world context, a similar interaction pattern might look like someone immediately closing online medical instructions, abandoning a digital intake form before completion, or stopping after initially encountering information that seems confusing or overwhelming. This interpretation also aligns with health informatics and digital health literacy research demonstrating that dense text, complex navigation demands, and limited digital literacy skills can create substantial barriers to engagement with online health information and patient portals [18,55].

The Hesitant-Minimal profile may reflect a different form of difficulty during engagement with the MEDCO task. Although these adults remained in the environment longer than the Early Exit group, they demonstrated relatively limited interaction with the information itself. Prior research on digital health literacy and patient portal usability suggests that adults with limited literacy skills often have trouble navigating online health environments, interpreting medical terminology, and identifying which information is most relevant to their immediate goals [55,56]. An everyday example of this pattern might look like someone repeatedly rereading dosage instructions, pausing over unfamiliar medical terminology, or spending time trying to determine which part of a health document contains the information needed to proceed. This state of hesitant persistence may emerge when dense medical language and unfamiliar digital navigation demands create sustained cognitive burden without facilitating efficient task progress [56,57].

In contrast, the Rapid Exploratory profile demonstrated the highest action rate within a moderate amount of time and relatively limited latency before interacting with the environment. This pattern may be consistent with moving quickly through the task, although the available process indicators cannot show how participants were reading or evaluating the information. Prior work suggests that rapid scanning of health-related materials may reduce deeper comprehension and limit integration of information with prior knowledge or task goals [58]. This interpretation is also consistent with broader digital health literacy research suggesting that users frequently prioritize speed, concise information, and rapid navigation when interacting with online health platforms, particularly within complex or information-dense environments [59]. Thus, frequent interaction was not necessarily associated with higher accuracy on this task.

Finally, within the primary five-profile model, the Direct/Routine and Deliberate Exploratory profiles demonstrated comparatively stronger performance outcomes despite exhibiting distinct patterns of engagement with the health-related materials. The Direct/Routine profile may resemble adults who carefully read prescription instructions, selectively identify key details within written health information, and briefly pause before responding to confirm relevant information. Eye-tracking research examining engagement with clinical information similarly suggests that sustained visual attention and reduced skimming behaviors may support more accurate retrieval of clinically relevant details during reading tasks [58]. In contrast, the Deliberate Exploratory profile may reflect adults who carefully compare dosage recommendations across sections of a document, revisit symptom information before making decisions, or return to previously read material to confirm that important details have not been overlooked. Importantly, both profiles were associated with relatively high accuracy, indicating that higher task accuracy occurred across more than one observed interaction pattern. This pattern is consistent with the possibility that successful performance on this task may be less tied to a single interaction style than to avoiding rapid disengagement or overly superficial processing, although the process data cannot determine this directly. This interpretation is also consistent with prior eHealth literacy research suggesting that effective use of digital health resources often requires users to move beyond surface-level interaction with digital interfaces and devote greater attention to evaluating relevant health information [54].

### 4.2. Heterogeneity in Demographics, Literacy Practices, and Task Performance

Beyond the distinct behavioral patterns observed across the engagement profiles, the broader demographic and literacy-related trends associated with these profiles were also consistent with prior research linking literacy, digital engagement, and health-related outcomes. Profiles characterized by stronger engagement and higher accuracy, particularly the Direct/Routine and Deliberate Exploratory groups, demonstrated higher educational attainment, greater reading and numeracy skills-use at home, and stronger overall literacy performance. This interpretation aligns with Feinberg et al. [53], who conceptualize literacy practices such as reading, writing, and engagement with functional materials as mutually reinforcing behaviors embedded within everyday domains including healthcare. Adults who routinely engage with reading, written materials, and numeracy-related tasks may therefore enter digital health contexts with greater familiarity interacting with complex information environments and sustaining engagement with cognitively demanding health materials.

The identified profiles also demonstrated descriptive demographic variation, particularly with respect to age and digital skills-use practices. The Early Exit profile included a somewhat larger proportion of older participants and demonstrated lower ICT skills-use overall, whereas the Rapid Exploratory profile included the largest proportion of younger participants and higher self-rated health ratings. These patterns are broadly consistent with prior digital health literacy research demonstrating that older adults and individuals with lower digital familiarity often encounter greater barriers when navigating digitally mediated healthcare systems [16,60,61]. At the same time, the relatively younger age and higher behavioral activity observed in the Rapid Exploratory group suggest that greater digital familiarity alone may not necessarily support more accurate or efficient engagement with health-related information. Rather, adults may enter digital health environments with different combinations of literacy practices and digital experiences.

The present findings suggest that engagement with a digitally presented health-related literacy task may be more behaviorally heterogeneous than aggregate performance outcomes alone imply. Research on digital health literacy has frequently relied on self-report measures, overall performance outcomes, or broad indicators of digital access when examining disparities in engagement with online health information [54]. Although this work has consistently documented barriers experienced by adults with lower literacy and digital skills, such approaches often provide limited insight into how those barriers unfold during real-time interaction with digital health environments. In contrast, process data can provide a more direct window into how adults behaviorally engage with digitally presented health-related information as interaction unfolds [38,39,40,43]. Importantly, the present findings suggest that observable engagement with the digital environment did not map neatly onto overall literacy outcomes or successful task completion. Higher levels of interaction and persistence within the environment did not necessarily correspond to successful performance, but they may nevertheless reflect ongoing attempts to work through the task, monitor information, or determine how to navigate the digital interface itself. These findings align with broader literacy research demonstrating that adults performing at or below proficiency thresholds do not represent a behaviorally homogeneous population [3,62]. Rather than supporting a simple deficit-oriented interpretation of task performance, the present findings suggest that participants with similar literacy outcomes may nevertheless engage with the same digitally presented health-related information in different ways that are not fully captured through aggregate scores or proficiency classifications alone.

### 4.3. Educational Implications

The behavioral complexity observed in the present study suggests that, in tasks like MEDCO, literacy and digital demands may interact rather than operate as entirely separate sources of difficulty. Although the MEDCO task involved a relatively constrained digital environment, successful engagement nevertheless required participants to sustain attention to digitally presented health information, identify task-relevant details, interpret written medical information, and appropriately interact with the digital interface itself. This perspective aligns closely with broader frameworks of literacy emphasizing that engagement within informational environments is shaped by the ways individuals respond to the demands of the task and the structure of the surrounding environment [19].

Guidance from adult digital literacy initiatives has similarly emphasized that effective participation in technology-rich environments requires more than procedural technology skills alone and is often most effective when digital skills instruction is embedded within meaningful real-world contexts and tasks [63,64,65]. For example, instruction may involve guided practice identifying task-relevant information within dense medical documents, modeling how to distinguish essential details from less relevant information, or supporting adults in more deliberately processing digitally presented health instructions [55]. Evidence further suggests that structured digital health literacy interventions can improve adults’ eHealth literacy, knowledge, and self-efficacy, particularly when instruction is sustained and supported through structured or theoretically guided learning opportunities [66,67].

Although the present study did not evaluate instructional supports, the observed engagement patterns raise hypotheses about where different forms of support may be useful. Findings from the present study, alongside prior process data research demonstrating that interaction behaviors and action sequences vary across performance groups during digital task engagement [39,40], suggest the value of future research examining supports that address both literacy and technology demands. From a literacy perspective, some learners may benefit from clearer organization and presentation of health information, scaffolds that support interpretation of medical terminology, and guidance in identifying and evaluating task-relevant content. From a technology perspective, learners may similarly benefit from supports that reduce unnecessary interface demands and provide clearer guidance for navigating unfamiliar digital environments, particularly given evidence that digital proficiency can vary across different forms of computer interaction and interface tasks among adults with developing literacy skills [68]. These possibilities should be viewed as directions for future instructional work rather than evidence that any particular support would be effective for the profiles identified here.

Within the context of the MEDCO environment, the present findings raise important questions about the extent to which different patterns of interaction may reflect varying challenges associated with digital interfaces, health-related content, or some combination of the two. In the item, participants were required to identify and highlight relevant information within the medical instructions, meaning that successful performance depended not only on interpreting the health information correctly but also understanding how to appropriately interact with the digital interface itself. For example, the Rapid Exploratory profile demonstrated frequent interaction with the environment but only moderate accuracy overall, suggesting that some may have had difficulty determining which information was relevant while others may have struggled with how the highlighting feature itself should be used to complete the task. In contrast, the Early Exit profile may reflect adults who disengaged almost immediately after entering the environment, potentially because instructions to “highlight” information required digital interaction skills that were not fully automatic, such as understanding how to click, drag, or select portions of text within the interface. Although the present data cannot definitively determine why these behaviors occurred or fully disentangle interface-related barriers from broader literacy difficulties, the findings nevertheless demonstrate how process data can help identify points during task engagement where different forms of difficulty may emerge. These patterns may, in turn, help generate hypotheses about where more targeted instructional support could be useful.

### 4.4. Limitations and Future Directions

A few limitations should be considered when interpreting our findings. First, the stability and generalizability of the identified profile solution warrant further examination. Sensitivity analyses showed that the exact number and composition of profiles varied across alternative indicator specifications. Although broader patterns of early exit, hesitant/minimal engagement, routine engagement, and exploratory engagement were recurrent, some finer distinctions, particularly between Rapid Exploratory and Deliberate Exploratory, were more sensitive to how action speed and latency were represented. The five profiles should therefore be interpreted as useful descriptive patterns identified under the primary model rather than as fixed or invariant behavioral types. The primary LPA was also estimated without sampling weights because the goal was to identify engagement patterns within the analytic sample rather than estimate their population prevalence. A sensitivity analysis incorporating the PIAAC final respondent sampling weight recovered the same five engagement patterns with highly similar classifications, but the primary profile proportions should still be interpreted as characteristics of the analytic sample rather than estimates of prevalence among U.S. adults. In addition, the analytic sample excluded 43 respondents with incomplete process data who had lower average literacy proficiency than participants retained in the analysis, which may have reduced representation of adults with particularly low literacy proficiency.

Second, the behavioral indicators available in the public use PIAAC Cycle 2 files provide relatively broad representations of participants’ interactions with the task. Although the MEDCO data captured actions, highlighting, and timing, full sequential organization of behavior throughout task completion was not captured. More fine-grained action sequence data could provide richer information about the order and timing of interactions and whether participants repeatedly selected information, shifted between sections of the instructions, or returned to previously viewed material [41,69]. Relatedly, these behavioral indicators cannot fully explain why a particular interaction pattern occurred. Similar behaviors could reflect difficulty using the interface, uncertainty about which information was relevant, or other underlying processes. Future research integrating more detailed process data with eye tracking, think-aloud protocols, and other observational approaches could help distinguish among these possibilities [70].

Third, the present study focused on a single PIAAC literacy item situated within a health-related context. Although PIAAC literacy tasks are intentionally designed to reflect everyday informational demands [9], MEDCO represents only one example of interaction with digitally presented health information. It therefore remains unclear whether the identified profiles reflect patterns that would recur across health-related tasks or whether some behaviors were specific to features of this item. Accordingly, the profiles should be interpreted as patterns of engagement with this specific health-related literacy task rather than as general profiles of digital health literacy. Future investigations using multiple comparable health-related tasks could help determine whether similar patterns emerge across task environments. Richer collections of repeated tasks could also support more advanced sequence-based approaches, such as multilevel hidden Markov models, for distinguishing recurring behavioral states from patterns that are more task specific [39,71].

Finally, MEDCO represents a relatively constrained digital environment because it was developed within the PIAAC Cycle 2 literacy domain rather than the more open-ended adaptive problem-solving (APS) domain [9]. We nevertheless draw on broader problem-solving frameworks such as RESOLV because successful performance required participants to identify relevant information, interpret instructions, and determine how to respond within a digital environment [19]. Real-world digital health environments often involve broader navigation and problem-solving demands, including coordinating information across screens and determining how to interact with unfamiliar systems. Although the PIAAC APS domain captures many of these broader digital problem-solving demands, its tasks do not appear to substantially represent healthcare-specific contexts. Future assessment development may therefore benefit from more ecologically valid digital health tasks that capture the combined literacy and problem-solving demands adults encounter when managing health information in digital settings.

## 5. Conclusions

Digital environments increasingly shape how adults access medications, interpret medical instructions, communicate with providers, and make everyday health decisions. Yet, the present findings suggest that participants did not engage with the MEDCO task in a uniform way. Instead, participants navigated the same task through multiple patterns of engagement. Some participants disengaged almost immediately, others remained hesitant and minimally interactive, whereas others actively explored the environment with varying levels of accuracy. At the same time, stronger performance emerged through more than one form of engagement, suggesting that there may not be a single “correct” observable interaction pattern associated with successful performance on this type of digitally presented health-related task. Rather, very low levels of interaction were associated with poorer task performance, whereas stronger performance occurred across more than one observed interaction pattern. As healthcare systems continue to expand reliance on patient portals, telehealth, and digitally mediated communication, process data may offer an important avenue for examining how different patterns of interaction unfold in digital health contexts.

## Figures and Tables

**Table 1:** Demographic and Literacy Characteristics of the Analytic Sample.

Variable and Category	*N*	%
Gender		
Female	1986	53%
Male	1736	47%
Age		
Younger (≤24 & 25–34)	1341	36%
Middle (35–44)	793	21%
Older (45–54 & 55+)	1588	43%
Health Status		
Excellent	627	17%
Very good	1178	32%
Good	1101	30%
Fair	633	17%
Poor	179	5%
Don’t Know/Refused	4	0.10%
Education		
HS or less	1759	47%
Some college	667	18%
BA or higher	1296	35%
Employment		
Employed	1896	51%
Unemployed	202	5%
Other, Don’t Know, Refused	1624	44%
Literacy Levels		
Below Level 1	373	11%
Level 1	613	16%
Level 2	1065	28%
Level 3	1176	32%
Level 4	462	12%
Level 5	33	1%

Note. HS = High School. BA = bachelor’s degree. Demographic frequencies are based on the analytic sample (*N* = 3722). Literacy proficiency distributions are weighted using PIAAC Cycle 2 sampling weights and therefore represent nationally representative estimates of the U.S. adult population.

**Table 2:** Model Fit Indices for the Primary Five-Indicator Latent Profile Analyses.

Profiles	Log-Likelihood	AIC	BIC	ICL	Entropy	*n* Min	*n* Max	*n* Min Proportion
3	−23,686.00	47,415.92	−47,552.80	−48,336.80	0.812	221	2045	0.059
4	−22,987.80	46,031.6	−46,205.80	−46,989.70	0.845	219	2104	0.059
**5**	**−22,625.30**	**45,318.67**	**−45,530.20**	**−46,554.50**	**0.830**	**168**	**1857**	**0.045**
6	−21,741.20	43,562.43	−43,811.30	−44,711.10	0.865	10	1638	0.003
7	−16,454.60	33,001.28	−33,287.50	−34,324.40	0.857	5	1729	0.001
8	−15,805.50	31,714.97	−32,038.50	−32,946.30	0.882	2	1660	0.001
9	−15,676.50	31,468.92	−31,829.80	−32,937.00	0.864	3	1499	0.001
10	−15,505.80	31,139.63	−31,537.80	−32,699.50	0.863	3	1419	0.001

Note. *n* min and *n* max indicate the sizes of the smallest and largest latent profiles, respectively, and *n* min proportion indicates the proportion of the analytic sample (*N* = 3722) contained in the smallest profile. Log-likelihood = model log-likelihood; AIC = Akaike Information Criterion; BIC = Bayesian Information Criterion; ICL = Integrated Completed Likelihood. For BIC and ICL values produced by mclust, larger values indicate better fit, including when values are negative; lower AIC values indicate better fit. Entropy ranges from 0 to 1, with higher values indicating greater classification precision. Candidate solutions were required to have entropy ≥ 0.75 and a minimum profile proportion ≥ 0.03; among solutions meeting these criteria, the solution with the largest BIC was preferred. Bold indicates the selected model.

**Table 3:** Descriptive Summary of Process Indicators by Profile.

Variable	Early Exit	Hesitant-Minimal	Rapid Exploratory	Direct/Routine	Deliberate Exploratory
*N*	206	722	168	1857	769
Actions	0.09 (0.36)	1.88 (0.95)	43.80 (30.94)	6.45 (2.96)	20.09 (10.48)
Total Time	7657.77 (14,989.21)	36,441.20 (22,005.89)	53,759.12 (35,726.40)	57,089.05 (30,950.62)	80,228.59 (42,125.54)
Visits	1.02 (0.17)	1.01 (0.09)	1.01 (0.08)	1.01 (0.09)	1.05 (0.26)
Latency Share	0.01 (0.07)	0.85 (0.10)	0.32 (0.15)	0.54 (0.15)	0.34 (0.15)
Action Speed	0.03 (0.18)	0.08 (0.08)	1.56 (7.37)	0.14 (0.10)	0.29 (0.14)
% Item Correct	0.00	0.30	0.51	0.58	0.62

Note. Values represent profile-specific means with standard deviations shown in parentheses. These values are reported on the original measurement scales and correspond to the standardized indicators displayed in Figure 2. Total Time is reported in milliseconds. Latency Share represents the proportion of total item time elapsed before participants had initiated interaction with the task, and Action Speed reflects the average number of actions performed per second during task engagement. % Item Correct represents the proportion of participants within each profile who answered MEDCO Question 2 correctly.

**Table 4:** Demographics by Profile.

Variables and Categories	Early Exit	Hesitant-Minimal	Rapid Exploratory	Direct/Routine	Deliberate Exploratory
Age					
Younger (≤24 & 25–34)	56 (27.8%)	249 (38.4%)	85 (56.8%)	675 (39.3%)	276 (38.3%)
Middle (35–44)	56 (29.0%)	145 (18.7%)	28 (14.5%)	399 (21.4%)	165 (21.0%)
Older (45–54 & 55+)	94 (43.2%)	328 (42.9%)	55 (28.6%)	783 (39.3%)	328 (40.7%)
Health Status					
Excellent	32 (14.6%)	114 (15.8%)	33 (19.4%)	292 (16.4%)	156 (21.6%)
Very good	46 (22.5%)	224 (32.0%)	59 (35.1%)	620 (33.2%)	229 (31.0%)
Good	69 (33.7%)	218 (32.1%)	40 (24.5%)	550 (29.4%)	224 (28.1%)
Fair	44 (22.1%)	130 (16.4%)	27 (15.7%)	307 (16.7%)	125 (15.7%)
Poor	14 (6.8%)	36 (3.8%)	9 (5.3%)	87 (4.3%)	33 (3.5%)
Don’t Know/Refused	1 (0.2%)	0 (0.0%)	0 (0.0%)	1 (0.0%)	2 (0.1%)
Education					
HS or less	159 (80.0%)	390 (54.1%)	84 (47.0%)	794 (44.4%)	332 (44.5%)
Some college	16 (7.6%)	123 (18.3%)	32 (22.7%)	352 (19.7%)	144 (18.7%)
BA or higher	31 (12.4%)	209 (27.5%)	52 (30.3%)	711 (35.9%)	293 (36.9%)

Note. HS = High School. BA = bachelor’s degree. Values are presented as *n* (%). Frequencies are unweighted observed counts, and percentages are survey-weighted within-profile estimates using the PIAAC final respondent sampling weight (SPFWT0). Percentages may therefore not correspond directly to the unweighted frequencies. Age categories were collapsed into younger (18–34 years), middle-aged (35–44 years), and older (45+ years) groups. Educational attainment categories were collapsed into high school or less (ISCED 0–3), some college or sub-baccalaureate education (ISCED 4–5), and bachelor’s degree or higher (ISCED 6–8).

**Table 5:** Home Skills-Use Practices and Literacy Proficiency by Latent Engagement Profile.

Variable	Early Exit	Hesitant-Minimal	Rapid Exploratory	Direct/Routine	Deliberate Exploratory
**Skills-Use**					
**ICT**					
*M* (*SD*)	1.62 (1.08)	2.32 (1.07)	2.30 (0.97)	2.35 (1.02)	2.33 (1.00)
95% CI	[1.42, 1.82]	[2.22, 2.42]	[2.13, 2.47]	[2.27, 2.42]	[2.23, 2.43]
**Numeracy**					
*M* (*SD*)	1.93 (1.36)	2.47 (1.09)	2.49 (1.02)	2.52 (0.92)	2.65 (0.90)
95% CI	[1.67, 2.19]	[2.37, 2.57]	[2.31, 2.67]	[2.45, 2.58]	[2.56, 2.74]
**Reading**					
*M* (*SD*)	1.84 (1.84)	2.67 (1.34)	2.66 (1.38)	2.81 (1.26)	2.87 (1.08)
95% CI	[1.55, 2.14]	[2.55, 2.79]	[2.39, 2.94]	[2.73, 2.88]	[2.79, 2.95]
**Writing**					
*M* (*SD*)	1.68 (1.26)	2.14 (1.07)	2.25 (0.95)	2.21 (0.98)	2.35 (0.87)
95% CI	[1.47, 1.90]	[2.03, 2.25]	[2.11, 2.40]	[2.15, 2.26]	[2.28, 2.42]
**Literacy Score**					
*M* (*SE*)	144.79 (4.90)	245.38 (2.66)	259.16 (7.18)	271.36 (1.62)	278.15 (2.57)
95% CI	[135.18, 154.40]	[240.18, 250.59]	[245.09, 273.23]	[268.19, 274.53]	[273.12, 283.19]

Note. *M* = Mean. *SD* = Standard Deviation. *SE* = Standard Error. *CI* = Confidence Interval. ICT = Information and Communications Technology. Higher values on the ICT, numeracy, reading, and writing indices indicate more frequent use of these skills at home. Skills-use estimates were survey weighted using the PIAAC final respondent sampling weight (SPFWT0), with confidence intervals estimated using the 80 replicate weights (SPFWT1–SPFWT80) and Fay’s balanced repeated replication procedures. Literacy proficiency estimates incorporate all 10 PIAAC literacy plausible values, the final respondent sampling weight, and the 80 replicate weights.

**Table 6:** Number and Percentage of Adults in Literacy Levels by Profile.

Literacy Level	Early Exit	Hesitant-Minimal	Rapid Exploratory	Direct/Routine	Deliberate Exploratory
Below L1 (<176)	77.8	14.4	11.5	4.9	2.7
L1 (176–225)	15.5	23	12.9	14.5	12.9
L2 (226–275)	5.1	29.3	33.3	30.4	30
L3 (276–325)	1.4	24.1	26.1	35.2	37.6
L4 (326–375)	0.6	7.8	15.1	13.6	15
L5 (≥376)	0.0	1.3	1.0	1.5	1.8

Note. Values represent survey-weighted literacy proficiency percentages within the groups defined by the primary model’s profile assignments. Percentages were estimated using the final respondent weight (SPFWT0) and averaged across the 10 PIAAC literacy plausible values (PVLIT1–PVLIT10). Because profile estimation itself was unweighted, these estimates should not be interpreted as estimates of the population prevalence of the latent profiles.

## Data Availability

Data is publicly available at the following website: https://www.oecd.org/en/data/datasets/PIAAC-2nd-Cycle-Database.html (accessed on 10 September 2025).

## Supplementary Materials

The following supporting information can be downloaded at: https://www.mdpi.com/article/10.3390/healthcare14203387/s1, Supplementary Text S1: Interpretation of Latent Profile Sensitivity Analyses Table S1: Sensitivity Analyses of Alternative Latent Profile Indicator Specifications; Table S2: Distribution of Number of Visits to the MEDCO Item; Table S3: Robustness of Survey-Weighted RQ2 Descriptive Results to Posterior-Probability Weighting.
