# Supplementary material for: Many Ways to Engage: Uncovering How Adults Navigate a Digitally Presented Health-Related Literacy Item
Source: Healthcare (Basel). 2026 Oct 11;14(20):3387. doi: 10.3390/healthcare14203387 (PMC13704261; doi:10.3390/healthcare14203387)
Supplement: Supplementary file 1 [file healthcare-14-03387-s001.zip › healthcare-4495411-supplementary.pdf]

Supplementary Materials

Supplementary Text S1. Interpretation of Latent Profile Sensitivity Analyses

Sensitivity analyses were conducted to evaluate whether the primary five-profile solution depended strongly on the inclusion or coding of particular process indicators (see Table S1). These analyses were interpreted using several sources of information rather than requiring exact replication of the primary classification. For each specification, we considered whether the solution met the prespecified entropy and minimum profile size criteria, the number and substantive characteristics of the resulting profiles, and correspondence with the primary classification using the Adjusted Rand Index (ARI). ARI values closer to 1 indicate greater agreement between classifications, whereas values near 0 indicate little agreement beyond chance. Because alternative indicator specifications can legitimately alter the number of profiles identified, ARI was interpreted as a continuous measure of correspondence rather than against a fixed cutoff.

Action speed and visits. Removing action speed favored a four-profile solution with relatively high correspondence to the primary classification (ARI = .78). The Early Exit, Hesitant-Minimal, and Direct/Routine profiles remained largely intact, whereas Rapid Exploratory and Deliberate Exploratory largely combined. This pattern suggests that interaction pace contributed substantially to distinguishing the two exploratory profiles, while the broader low-engagement, hesitant, routine, and exploratory patterns remained recognizable.

Removing visits favored a seven-profile solution, although this appeared to reflect additional subdivision rather than the emergence of substantively different engagement patterns. The preferred seven-profile solution had relatively low correspondence with the primary classification (ARI = .42), but an admissible five-profile solution under the same specification showed substantially greater correspondence (ARI = .78), with 89%–100% of each primary profile retained. Because 98.3% of participants visited the item only once (Table S2), these results suggest that visits were not necessary to recover the primary five engagement patterns, although their inclusion affected the degree of subdivision preferred by the model.

When both action speed and visits were removed, the preferred solution contained six profiles and correspondence with the primary classification was lower (ARI = .56). An admissible five-profile solution remained identifiable, with moderate correspondence to the primary model (ARI ≈ .60). Most of the broader engagement structure remained recognizable, but the Rapid Exploratory pattern was less stable. Taken together, these analyses suggest that action speed contributed more strongly than visits to the distinction between the exploratory profiles.

Latency specifications. Removing latency share entirely again produced a five-profile solution and recovered the same broad substantive engagement patterns (ARI = .62). Correspondence was not exact, particularly for Hesitant-Minimal, but retention of participants within corresponding profiles ranged from approximately 70% to 95%. Thus, latency influenced individual profile assignments but was not necessary for the five-profile structure to emerge.

Results were more sensitive to how latency was represented for participants with no recorded actions. When the entire item interval for these participants was treated as occurring before an action, yielding a latency share of 1 rather than the public-use value of 0, a four-profile solution was preferred (ARI = .61). Hesitant-Minimal, Direct/Routine, and Rapid Exploratory remained highly recognizable, and most Early Exit participants remained in a distinct low-engagement group, whereas Deliberate Exploratory was divided across routine and exploratory groups. This suggests that the fifth profile distinction was more sensitive to the treatment of no-action latency than the broader engagement structure.

Replacing latency share with initiation or exit time again yielded five profiles, but correspondence with the primary classification was low (ARI = .27). This finding indicates that recovering the same number of profiles should not by itself be considered evidence of replication when the composition and substantive patterning of those profiles differ. Finally, the raw first action latency analysis among participants who recorded an action produced no solution meeting the prespecified criteria and excluded the no-action behavior central to the Early Exit profile, making it less directly comparable with the full sample analyses.

Sampling-weighted model. Re-estimating the primary five-indicator model using the PIAAC final respondent sampling weight produced the same five substantive engagement patterns with very high correspondence to the primary classification (ARI = .92; 96.7% corresponding classifications). This provides evidence that the primary profile structure was not driven by estimating the LPA without the survey sampling weight.

Overall interpretation. Across sensitivity analyses, the broader patterns of early exit, hesitant/minimal engagement, routine engagement, and exploratory engagement were recurrent. The primary five-profile solution was also recovered when latency was removed and in the sampling-weighted model. At the same time, the distinction between Rapid Exploratory and Deliberate Exploratory was more sensitive to indicator selection and latency coding than the broader profile structure. We therefore retained the primary five-profile solution while interpreting the two exploratory profiles as a finer distinction that warrants greater caution.

Table S1

Sensitivity Analyses of Alternative Latent Profile Indicator Specifications

Specification	Change from primary model	*N*	Preferred number of profiles	Entropy	Smallest profile, *n* (%)	ARI with primary classification	Revised interpretation relative to primary model
Primary specification	Actions, total time, visits, latency share, action speed	3,722	5	.830	168 (4.5%)	1.00	Five profiles were selected under the prespecified criteria for the full five-indicator model. The neighboring four-profile solution did not cleanly separate the Deliberate Exploratory pattern, whereas the six-profile solution largely retained the five patterns but added a 10-person extreme-response class.
No action speed	Action speed excluded; original latency coding retained	3,722	4	.818	222 (6.0%)	.78	Early Exit, Hesitant-Minimal, and Direct/Routine remained almost entirely intact, while Rapid Exploratory and Deliberate Exploratory largely combined. This suggests that the distinction between the two exploratory profiles depends substantially on action speed rather than indicating instability in the broader profile structure.
No visits	Visits excluded; original latency coding retained	3,722	7	.762	180 (4.8%)	.42	The preferred seven-profile solution reflected additional subdivision. Importantly, an admissible five-profile solution remained highly similar to the primary model (ARI = .78), with 89%–100% of each primary

							profile retained. Thus, visits were not necessary to recover the five primary patterns.
No action speed or visits	Both action speed and visits excluded; original latency coding retained	3,722	6	.753	219 (5.9%)	.56	Removing both indicators produced more restructuring, particularly for Rapid Exploratory. However, an admissible five-profile solution also remained, with moderate correspondence to the primary model (ARI ≈ .60). This suggests that most of the broader structure remained recognizable, but the finer exploratory distinction became less stable when both pace and revisiting information were removed.
No latency	Latency share excluded; actions, total time, visits, and action speed retained	3,722	5	.785	170 (4.6%)	.62	The same five substantive engagement patterns were recovered without a latency indicator. Retention of the primary profiles ranged from 69.7% for Hesitant-Minimal to 94.6% for Rapid Exploratory, indicating that latency influenced some individual assignments but was not necessary to recover the five-profile structure.
Alternative no-action latency coding	For participants with no recorded actions, the entire item	3,722	4	.849	176 (4.7%)	.61	Preferred four-profile solution retained 98% of Hesitant-Minimal, 95% of

	interval was treated as occurring before an action, yielding a latency share of 1 rather than the public-use value of 0.						Direct/Routine, and 92% of Rapid Exploratory participants in corresponding groups. Most Early Exit participants remained in a distinct low-engagement group, whereas Deliberate Exploratory split between the routine and exploratory groups. This supports four recurrent broader patterns but indicates that the fifth Deliberate Exploratory distinction is sensitive to no-action latency coding.
Initiation-or-exit latency	Latency share replaced by raw first-action latency for actors and task-exit time for non-actors	3,722	5	.779	120 (3.2%)	.27	Five profiles again met the selection criteria, but correspondence with the primary assignments was low (ARI = .27). Thus, recovering five profiles alone should not be viewed as replication; the composition of the profiles was sensitive to this substantially different representation of latency.
Raw first-action latency, action-takers only	Latency share replaced with raw first-action latency; zero-action respondents excluded	3,529	No eligible solution†	—	—	—	No solution met the prespecified criteria. This analysis also excludes respondents with no recorded actions, including the behavior central to the Early Exit profile, so it is not directly comparable to the full sample primary model.

Sampling-weighted model	Primary five indicators estimated using SPFWT0	3,722	5	.838	168 (4.5%; weighted 4.7%)	.92	The weighted analysis reproduced the same five substantive patterns with very high correspondence to the primary model (ARI = .92; 96.7% corresponding classifications), providing strong evidence that the primary structure was not driven by the omission of the sampling weight.

Note. ARI = Adjusted Rand Index. ARI summarizes agreement between two classifications while adjusting for agreement expected by chance; values closer to 1 indicate greater correspondence, whereas values near 0 indicate little agreement beyond chance. ARI was treated as a continuous index of correspondence rather than evaluated against a fixed cutoff and was interpreted together with the number and size of profiles and their substantive patterning. Except where otherwise noted, ARI compares the preferred solution under each sensitivity specification with the primary five-profile classification and can therefore compare partitions containing different numbers of profiles. For the no-visits specification, the preferred seven-profile solution had ARI = .42 with the primary solution; the alternative five-profile no-visits solution had ARI = .78. †No model containing three through ten profiles simultaneously satisfied entropy ≥ .75 and a minimum profile proportion ≥ .03. The initiation-or-exit latency specification treats task-exit time as an alternative representation of the period without an observed action for respondents who recorded no actions; it does not represent an observed first action for those respondents.

Table S2

Distribution of Number of Visits to the MEDCO Item

Number of visits	*n*	%
1	3,660	98.33
2	57	1.53
3	4	0.11
4	1	0.03
Total	3,722	100.00

Note. Values describe the unweighted analytic sample used for latent profile estimation.

Table S3

Robustness of Survey-Weighted RQ2 Descriptive Results to Posterior-Probability Weighting

Profile	Maximum demographic shift (pp)	Maximum skills-use mean shift	Modal literacy mean	Posterior-weighted literacy mean	Literacy mean change	Maximum literacy-level shift (pp)
Early Exit	0.19	0.010	144.79	144.52	−0.26	0.13
Hesitant-Minimal	1.01	0.016	245.38	247.78	+2.40	1.14
Rapid Exploratory	1.69	0.017	259.16	258.97	−0.19	1.40
Direct/Routine	0.36	0.012	271.36	271.09	−0.27	0.32
Deliberate Exploratory	1.04	0.033	278.15	276.83	−1.32	0.67

Note. Primary RQ2 estimates use most likely profile assignments and the PIAAC survey weights. The sensitivity estimates use posterior membership probabilities together with the survey weights, allowing participants to contribute to each profile according to their estimated probability of membership. Maximum demographic shift is the largest absolute percentage-point difference across age, education, and self-rated health categories. Maximum skills-use shift is the largest absolute difference across ICT, numeracy, reading, and writing means. Smaller absolute differences between the primary and posterior-probability-weighted estimates indicate greater robustness of the RQ2 descriptive findings to uncertainty in profile assignment. Literacy estimates incorporate all 10 plausible values, the final sampling weight, and the 80 replicate weights.
